# The Hospitals Association

**Published:** 1889-03-30

**Authors:** 


### THE HOSPITALS ASSOCIATION.

We are obliged to hold over Mr. Ryan's paper on " Street Ambulances," and the report of the highly-successful meeting at the Middlesex Hospital, until next week.

